# Modifiable predictors of mental health literacy in the educational context: a systematic review and meta-analysis

**DOI:** 10.1186/s40359-024-01878-4

**Published:** 2024-07-04

**Authors:** Charin Suwanwong, Anchalee Jansem, Ungsinun Intarakamhang, Pitchada Prasittichok, Sudarat Tuntivivat, Krittipat Chuenphittayavut, Khuong Le, Le Thi Mai Lien

**Affiliations:** 1 Behavioral Science Research Institute, Srinakharinwirot University, Bangkok, Thailand; 2 Faculty of Humanities, Srinakharinwirot University, Bangkok, Thailand; 3 Faculty of Psychology, University of Social Sciences and Humanities, Vietnam National University, Ho Chi Minh City, Vietnam

**Keywords:** Mental health literacy, Education, Predictors, Systematic review, Meta-analysis

## Abstract

Mental health literacy is vital for well-being in educational settings, extending beyond academics to include social and emotional development. It empowers individuals, allowing them to recognize and address their mental health needs and provide essential support to their peers. Despite the acknowledged importance of modifiable factors, there is a noticeable research gap in those amenable to change through educational interventions. Thus, this systematic review aims to identify potentially modifiable predictors of mental health literacy in the educational context. A systematic search was conducted for quantitative studies published between 2019 and October 2023 using several databases following PRISMA guidelines. Studies needed to focus on potentially modifiable predictors of mental health literacy in the educational context. Study quality was assessed using the Appraisal tool for Cross-Sectional Studies (AXIS tool). In total, 3747 titles and abstracts were screened, 60 articles were assessed in full-text screening, and 21 were included in the review. Significant correlations between mental health literacy and modifiable predictors, including stigma toward professional help, self-efficacy, attitudes toward help-seeking, social support, positive psychological states, receiving mental health training, and psychological distress, were identified. By addressing these factors, educational institutions can cultivate community’s adept in mental health, fostering an environment marked by empathy, understanding, and proactive engagement in addressing mental health issues. The implications serve as a foundation for future research, policy development, and implementing of practical strategies to enhance mental health literacy in diverse educational settings.

## Introduction

Mental health literacy (MHL), introduced by Australian researcher Anthony Jorm and colleagues in their influential 1997 work, is defined as ‘knowledge and beliefs about mental disorders that facilitate their recognition, management, or prevention [1]. This concept includes various components: (1) the ability to recognize specific disorders or types of psychological distress; (2) knowledge and beliefs about risk factors and causes; (3) knowledge and beliefs about self-help interventions; (4) knowledge and beliefs about available professional help; (5) attitudes that facilitate recognition and appropriate help-seeking; and (6) knowledge of how to seek mental health information [2]. MHL extends beyond mere conceptual comprehension of mental health issues; it involves knowledge aimed at increasing the likelihood of taking action to improve one’s own mental health or that of others [3]. This includes knowing how to attain and sustain good mental health, understanding mental disorders and their treatments, reducing associated stigma, and promoting effective help-seeking abilities, including knowing when, where, and how to access quality mental health care and developing competencies for self-care [4, 5]. Robust population-based research on MHL is actively progressing, offering valuable insights for mental healthcare systems and professionals as they adapt their practices to serve better individuals facing mental health challenges [6]. Furthermore, it’s essential to emphasize that MHL is contextually situated, often involving numerous stakeholders and is particularly relevant in developmental contexts [7, 8], especially within educational settings where young people and school staff represent primary targets.

In educational settings, where the cultivation and dissemination of knowledge take center stage, nurturing MHL assumes critical significance [9, 10]. It not only provides individuals, whether they are students, educators, or staff members, with essential tools for recognizing and addressing mental health challenges within themselves but also empowers them to be better prepared to offer support to their peers or students who may be grappling with mental health issues [11–13]. This interconnected support system not only alleviates feelings of isolation but also fosters an environment conducive to open dialogues surrounding mental health concerns [14, 15]. As a result, it fosters a culture marked by empathy and understanding within educational environments, thus facilitating the ability to assist others and the creation of a more inclusive, safe, and supportive learning atmosphere [16, 17].

Previous research on MHL has primarily centered on individuals, including students, teachers, educators, and various community members [18–20]. While this body of work has significantly contributed to our understanding of how individuals perceive and engage with mental health issues, it has often overlooked a crucial aspect—the MHL within the broader educational environment itself, which has faced unprecedented challenges due to the COVID-19 pandemic. Educational settings, whether schools, colleges, or universities, serve as multifaceted ecosystems where myriad interactions unfold daily. They are not merely places of academic learning but dynamic environments where social, emotional, and intellectual development intersect [21, 22]. In the wake of COVID-19, the significance of addressing mental health literacy within educational institutions has become even more pronounced, given the additional stressors and uncertainties faced by students, teachers, and the entire educational community [23, 24]. Therefore, examining and enhancing MHL within the educational context is imperative. This investigation addresses a noticeable void in current research, specifically the lack of focus on modifiable factors related to MHL that can be influenced through targeted educational interventions. While previous studies have delved into non-modifiable factors such as age, gender, and prior exposure to mental health issues [25–27], there is a significant research gap when it comes to comprehensively analyzing factors that can be modified and improved through intentional educational strategies.

By addressing this research gap, this systematic review aims to identify potentially modifiable predictors of MHL in the educational context. It emphasizes the critical importance of these modifiable predictors, providing actionable insights to guide the design of educational interventions aimed at enhancing MHL within educational institutions. By clarifying these modifiable predictors, this research provides a roadmap for educational institutions to customize interventions that address areas of MHL deficiency among students and faculty. These interventions have the potential to nurture a culture of understanding, empathy, and support for mental health issues within educational environments. In doing so, this research aims to make a meaningful contribution to the cultivation of healthier and more mentally literate educational environments.

## Methods

### Search strategy

In accordance with the guidelines outlined in the Preferred Reporting Items for Systematic Reviews and Meta-Analyses (PRISMA) [28], an extensive search was carried out to identify relevant articles published between 2019 and October 2023. A systematic search was conducted across global databases (PubMed, SCOPUS, PsycINFO and ERIC), as well as regional databases (TCI and Vietnamese Database), to capture a diverse range of studies relevant to MHL within educational contexts. This approach ensures comprehensive coverage while considering the specific geographical and cultural context of the research. These search terms included a combination of terms related to predictors, factors, associations, or relationships, along with terms related to MHL, which were developed following the conceptualization by Jorm [2] and educational contexts. Once the search terms were established, they were adapted to suit the specific search language of each respective database. The search criteria included the following terms: (predictors OR factors OR association OR relationship) AND (mental health literacy) AND (education OR school OR university OR students OR teacher OR professor).

### Eligibility criteria and study selection

The inclusion criteria were: (1) studies involving populations within an educational context, including but not limited to students (from primary to higher education levels), teachers, lecturers, professors, school administrators, counselors, and similar educational personnel; (2) studies containing quantitative data investigating potentially modifiable predictors related to MHL; (3) studies assessing MHL as an outcome measure using validated instruments or measures; and (4) studies published in peer-reviewed journals, available in English, Thai, or Vietnamese languages. The exclusion criteria were: (1) studies adopting a qualitative research design which may not provide suitable data for inclusion in a meta-analysis; (2) studies utilizing an experimental research design, which focuses on the intervention to enhance MHL; (3) studies with insufficient statistical data for meta-analysis, including missing and incomplete data on key variables relevant to the study’s objectives; or (4) studies omitting information about the educational institution of the participants, including details regarding the type of educational setting (e.g., primary school, university). Two authors (CS & PP) independently assessed the relevance of the study titles and abstracts to determine eligibility, and any disagreements were resolved through team discussion.

### Data extraction

Information retrieval during data extraction included details such as the first author’s name, year of publication, country of origin, study participants, sample size, the instruments used to assess MHL, and the main findings. Two authors (CS & AJ) independently conducted the data extraction process, and their findings were cross-checked and harmonized through discussions. Consensus was achieved through ongoing discussion within the research team.

### Quality assessment

The assessment of the quality of the included studies was conducted using the Appraisal tool for Cross-Sectional Studies (AXIS tool) [29]. This tool comprises 20 questions that address various aspects of study design, reporting quality, and the risk of bias in cross-sectional studies. A score of 1 was assigned for each ‘yes’ response to a question, while a score of 0 was given for ‘no’ or ‘don’t know’ responses. Based on the total score represented as a percentage, the studies were categorized into one of three groups: high quality (above 80%), moderate quality (between 60% and 80%), or low quality (below 60%). The quality assessment for each study was independently evaluated by two authors (CS & UI). In cases where discrepancies arose in the ratings between the two authors, these differences were resolved through team discussions.

### Statistical analysis

We conducted meta-analyses using effect sizes based on correlation coefficients between two continuous variables measuring MHL and the respective factor. The majority of the studies provided direct correlation coefficients (r). For those that presented different metrics, such as odds ratios, t-values, F-values, or standardized regression coefficient (β), we converted them into correlation coefficients. For this purpose, correlation coefficients are transformed into Fisher’s Z and estimated with 95% confidence intervals (CIs) using a random-effects model. To assess heterogeneity among the studies, we employed Cochrane’s Q test and I^2^ statistics [30]. Egger’s test was employed to assess the presence of publication bias. All statistical analyses were performed using RStudio software (v. 4.3.1), and the packages metafor [31].

## Results

### Characteristics of included studies

**Fig. 1:**
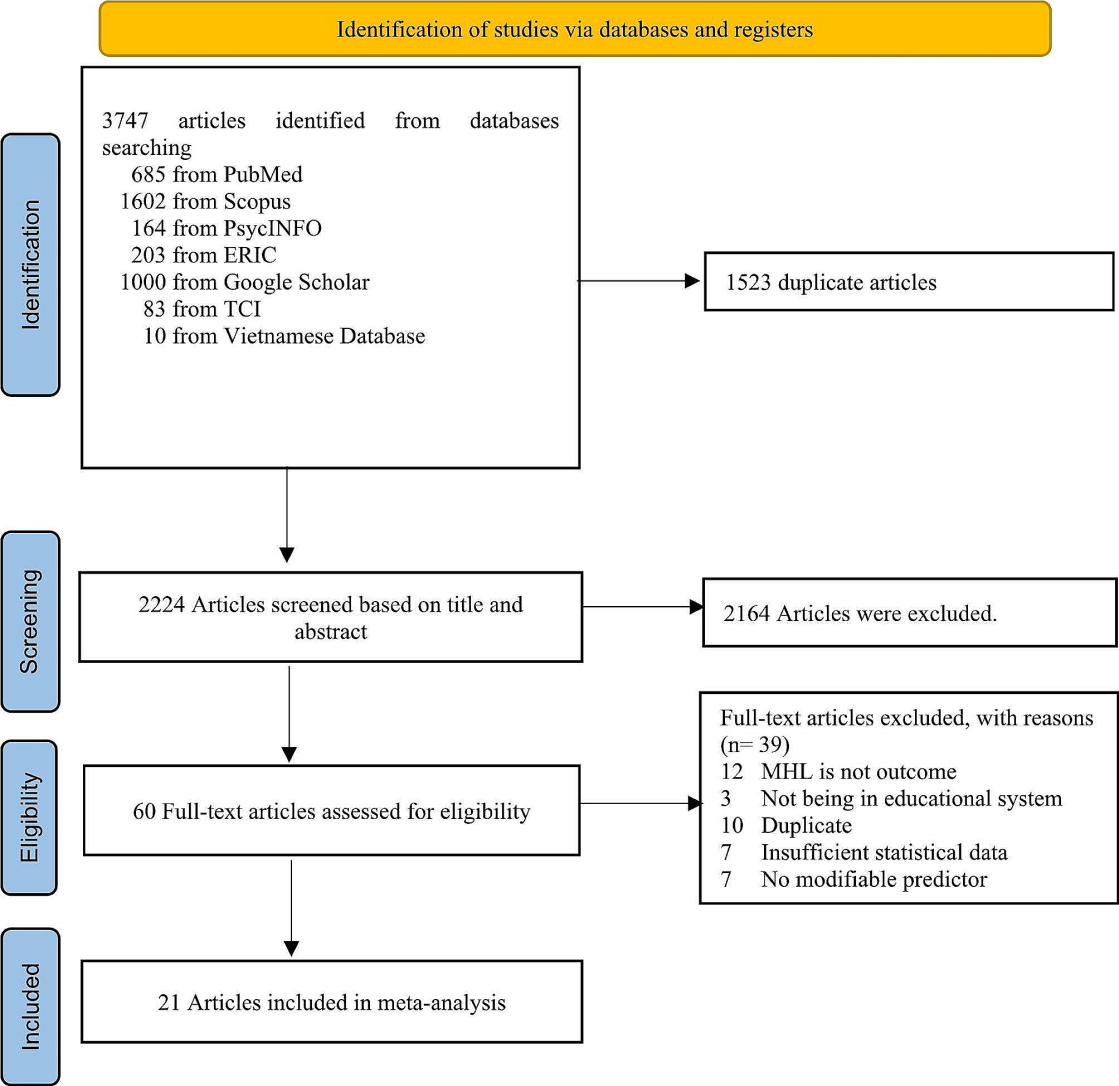
PRISMA diagram

In this systematic review, database searches were performed, revealing a total of 3747 articles. After removing duplicates, 2224 articles underwent screening, and 60 were subjected to full-text review. Finally, 21 studies met the eligibility criteria. Primary reasons for exclusion included studies not conducted in educational settings, inadequate statistical data, and non-modifiable variables. This process is summarized in Fig. 1.

The studies involved a diverse range of participants, including university students (*n* = 12), teachers (*n* = 5), secondary and high school students (*n* = 3), and a mix of university and secondary school students (*n* = 1). The majority of participants were female. Most studies utilized mental health literacy scale as outcomes. The majority of the research (*n* = 18) was conducted in middle-income countries like China, Malaysia, Taiwan, Thailand, and others. A small number of studies (*n* = 3) were carried out in both low- and high-income countries. Primary research settings included universities (*n* = 12), followed by secondary and high schools (*n* = 5), among others. All included studies underwent peer review, and their quality was assessed using the AXIS tool. Rating indicated high quality for 7 studies, moderate for 11 studies, and low for 3 studies. A summary of study characteristics is presented in Table 1.

**Table 1:** Characteristics of included studies

Author (Year)	Country	Educational setting	Participant characteristics	MHL measurement	Modifiable determinants of MHL	Quality assessment
Chen et al. (2021) [32]	**China**	**Primary and secondary school**	Teachers *n* = 367 89.6% female 67.8% no experience with mental illnesses	MHLS (Chinese)	Stigma for receiving psychological help Social distance Self-efficacy in coping with mental health problems	High
Salloum & Ismail (2020) [33]	Malaysia	University	University students *n* = 153 60.8% male 88.2% no experience with mental illnesses	MHLS	Being to a mental health professional	Low
Ibrahim et al. (2019) [34]	Malaysia	Secondary school and University	*n* = 202 All *n* = 127 secondary school students *n* = 75 university students 67.8% female	MHSAS	General help seeking Self-stigma of seeking help Negative belief toward mental illness	Moderate
Lee et al. (2023) [35]	Malaysia	Secondary school	Secondary school students *n* = 450 61.8% female	MHAS	Knowledge on mental health Knowledge on professional help Attitude towards mental health	Moderate
Singh et al. (2022) [36]	Malaysia	Secondary school	Secondary school students *n* = 1400 51.7% female 90.9% no experience with mental illnesses	MHLS	Health Behavior Bullying Loneliness	High
Seboka et al. (2022) [37]	Ethiopia	University	University students *n* = 780 62.6% male 90.9% no experience with mental illnesses	MHLS	Online mental health information-seeking behaviors Digital competence	Moderate
Lo et al. (2023) [38]	Taiwan	University	University students *n* = 568 64.0% female	MHLS	Perceived social support Mindfulness Hope	High
Mamun et al. (2020) [39]	Bangladesh	University	University students *n* = 404 62.6% male 73.8% have experience with mental illnesses	D-Lit	Attend any seminars, workshops, or other programs related to mental health Awareness any seminars, workshops, or other programs related to mental health	Moderate
Qian et al. (2023) [40]	China	Preschool	Teachers *n* = 2352 97.2% female	National Mental Health Literacy Questionnaire	Perceived stress Anxiety	Moderate
Lu et al. (2021) [41]	Taiwan	University	University students *n* = 1294 55.8% male	MHLS	Mental illness stigma attitude Help-seeking efficacy Maintenance of positive mental health	Moderate
Hsu et al. (2019) [42]	Taiwan	Preschool	Teachers *n* = 534 97.2% female	PTMHL	Online learning community Enthusiasm for engagement	Moderate
Abdelsalam & Said (2022) [43]	Egypt	University	University students *n* = 361 65.4% female 91.1% no experience with mental illnesses	D-Lit LOSS	Previous training on mental health	High
Prabhu et al. (2021) [44]	India	High school	Teachers *n* = 460 70.0% female	Australian National Mental Health Literacy and Stigma Youth Survey	Seek information on mental health Able to spread awareness Able to provide referral	Low
Pribadi et al. (2023) [45]	Indonesia	University	University students *n* = 650 67.7% female	MHLQ	-	Low
Dessauvagie et al. (2022) [46]	Vietnam Cambodia	University	University students *n* = 1041 51.0% male 84.5% no experience with mental illnesses	MHLS (Vietnamese)	-	Moderate
Sittironnarit et al. (2022) [47]	Thailand	University	University students *n* = 202 65.8% female	MHLS (Thai)	Seeking help from mental health professionals Having a mental health professional as an intimate contact	Moderate
Hu et al. (2021) [48]	China	University	University students *n* = 1334 74.7% female	National Mental Health Literacy Questionnaire	Stress of COVID-19 Economic insecurity	High
Marwood & Hearn (2019) [49]	United Kingdom	University	University students *n* = 251 66.9% female	MHLS	Undergone treatment for a mental illness	Moderate
Li et al. (2022) [50]	China	Primary school High school University	Teachers *n* = 573 76.1% female	MHLQ	Social support Life satisfaction Coping tendency	High
Wenzler & Keeley (2022) [51]	United States	University	University students *n* = 496 75.1% female	MMHL	Received mental health services	High
Chidmongkol et al. (2020) [52]	Thailand	High school	High school students *n* = 420 60.0% female	MHLS	Depression	Moderate

MHLS: Mental Health Literacy Scale; MHSAS: Mental Help Seeking Attitude; MHAS: Mental Health Awareness Scale; PTMHL: Preschool Teachers’ Mental Health Literacy Scales; D-Lit: Depression Literacy Scale; LOSS: Literacy of Suicide Scale; MHLQ: Mental Health Literacy Questionnaire; MMHL: Multicomponent Mental Health Literacy

### Modifiable predictors of MHL

In total of 21 studies, we identified more than 20 potentially modifiable predictors of MHL in an educational context. However, we specifically conducted random-effects meta-analyses for those potentially modifiable predictors reported in more than two studies to assess the strength of the association between these predictors and MHL. Any predictor mentioned in only one study was excluded from the pooled effect size analysis in this study, or if the reported data could not be converted to the effect size (*r*). Consequently, we performed eight meta-analyses to explore the association between modifiable predictors (Stigma toward professional help, stigma toward mental illness, self-efficacy, seeking help from mental health professionals, attitudes toward help-seeking, social support, positive psychological states, receiving mental health training, and psychological distress) and MHL within educational contexts (Table 2).

**Table 2:** Random-effect model of the modifiable predictors of mental health literacy

Modifiable predictors	*k*	*n*	Mean *r* effect size, random-effects model	95% Confidence Intervals		Test of null (two-tail)		Heterogeneity				Egger’s regression		
				Lower 95% CI	Upper 95% CI	Z	*p*-value	Q	df	*p*-value	I^2^	*t*	df	*p*-value
Stigma towards professional help	3	771	-0.33	-0.45	-0.19	-4.46	0.000	8.98	2	0.011	77.7	2.88	1	0.213
Stigma towards mental illness	3	1698	0.00	-0.13	0.14	0.06	0.956	10.30	2	0.006	80.6	-4.82	1	0.130
Self-efficacy	4	2581	0.24	0.18	0.30	7.94	0.000	6.64	3	0.085	54.8	-4.41	2	0.048
Seeking help from mental health professionals	4	1591	0.29	0.25	0.34	12.07	0.000	0.27	3	0.966	0.0	0.69	2	0.560
Attitudes towards help seeking	4	1388	0.30	0.00	0.55	1.99	0.047	120.29	3	0.000	97.5	-1.25	2	0.339
Social support	5	3196	0.41	0.05	0.68	2.21	0.027	748.26	4	0.000	99.5	-2.61	3	0.079
Positive psychological states	4	2243	0.37	0.30	0.43	10.04	0.000	9.75	3	0.021	69.2	1.32	2	0.318
Received mental health training	4	1530	0.33	0.18	0.47	4.13	0.000	31.57	3	0.000	90.5	1.49	2	0.274
Psychological distress	4	6399	-0.39	-0.47	-0.30	-8.33	0.000	45.56	3	0.000	93.4	0.58	2	0.618

k = Number of studies, n = Number of subjects, df = degree of freedom

### Stigma toward professional help

**Fig. 2:**
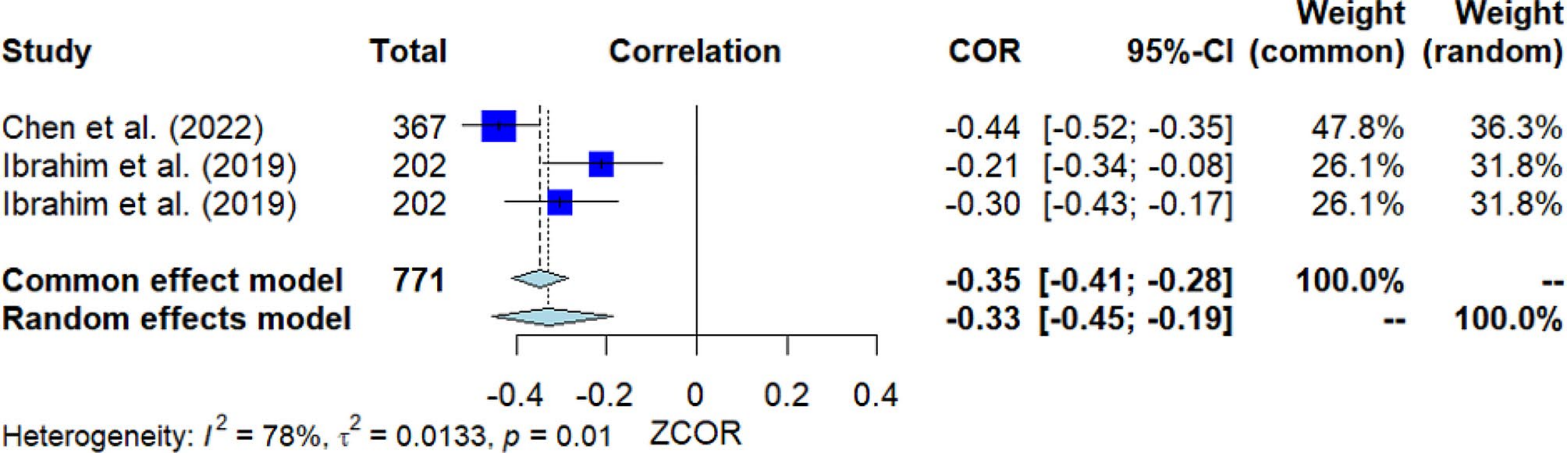
Forest plot for the correlation between stigma toward professional help and MHL

Three studies investigated the correlation between stigma toward professional help and MHL (Fig. 2). The pooled correlation resulted in a significant negative finding, with *r* = -0.33 [95% *CI*: -0.45, -0.19]. The *Q*-test revealed substantial heterogeneity among the studies (*Q*(2) = 8.98, *p* = 0.011, *I*^2^ = 77.7%). Nevertheless, Egger’s test did not show significance, indicating no presence of publication bias.

### Stigma toward mental illness

**Fig. 3:**
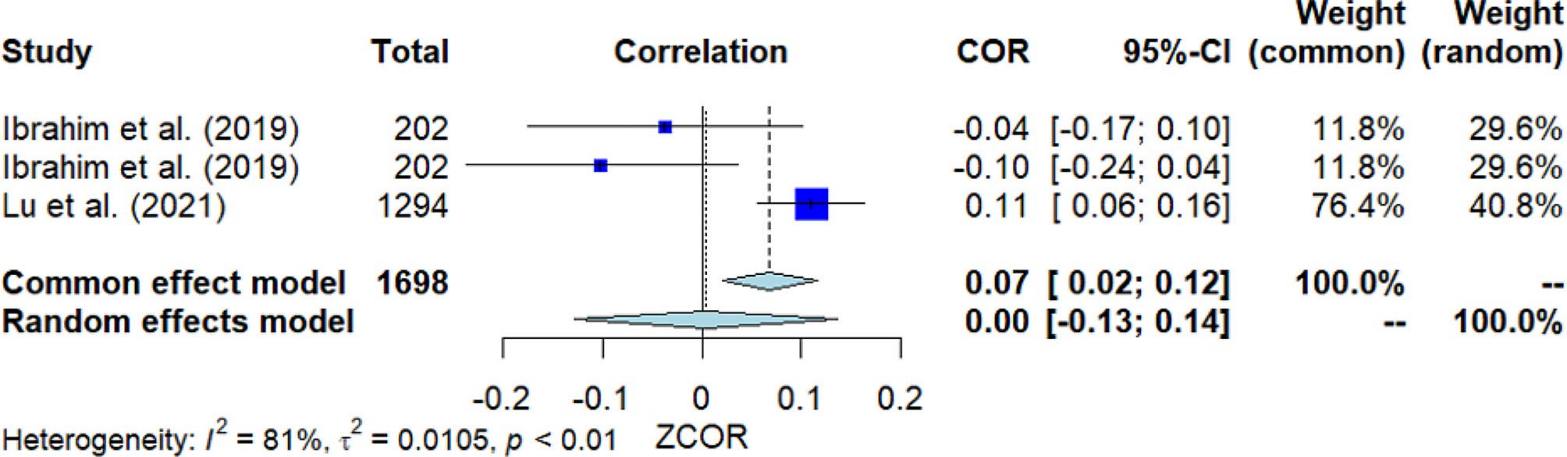
Forest plot for the correlation between stigma toward mental illness and MHL

Three studies investigated the correlation between stigma toward mental illness and MHL (Fig. 3). The pooled correlation produced a non-significant outcome, with *r* = 0.00 [95% *CI*: -0.13, 0.14]. The *Q*-test revealed significant heterogeneity among the studies (*Q*(2) = 10.30, *p* = 0.006, *I*^2^ = 80.6%). However, Egger’s test did not show significance, indicating no presence of publication bias.

### Self-efficacy

**Fig. 4:**
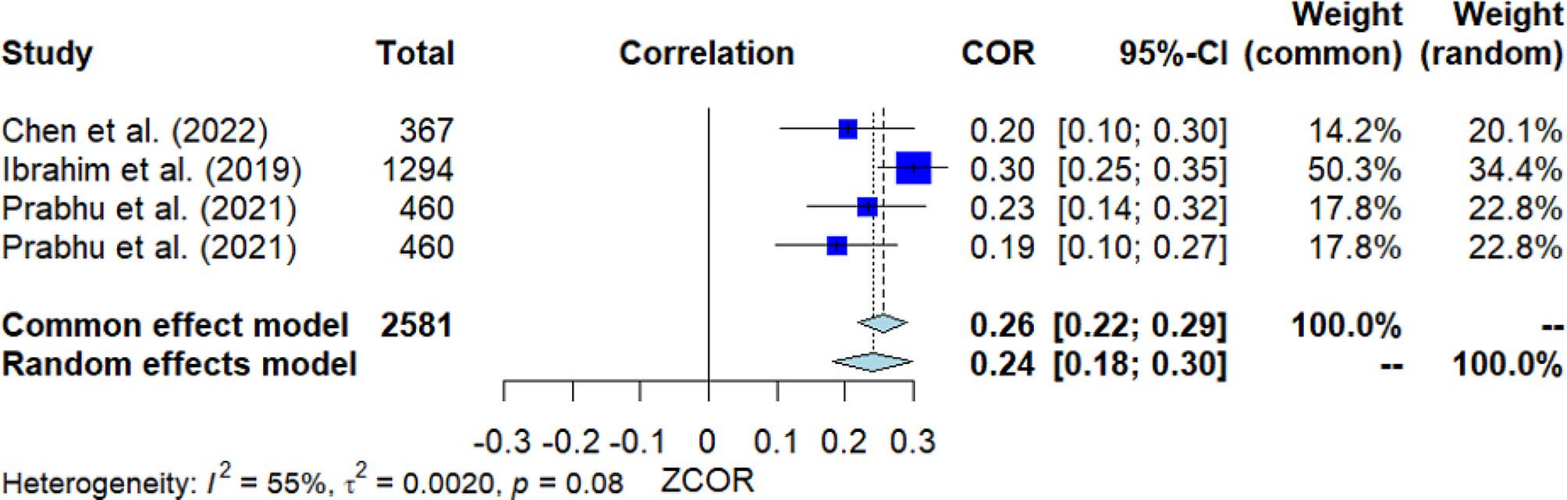
Forest plot for the correlation between self-efficacy and MHL

Four studies investigated the correlation between self-efficacy and MHL (Fig. 4). The pooled correlation resulted in a significant positive finding, with *r* = 0.24 [95% *CI*: 0.18, 0.30]. The *Q*-test showed no heterogeneity among the studies (*Q*(3) = 6.64, *p* = 0.085, *I*^2^ = 54.8%). However, Egger’s test indicated significance, suggesting the potential presence of publication bias.

### Seeking help from mental health professionals

**Fig. 5:**
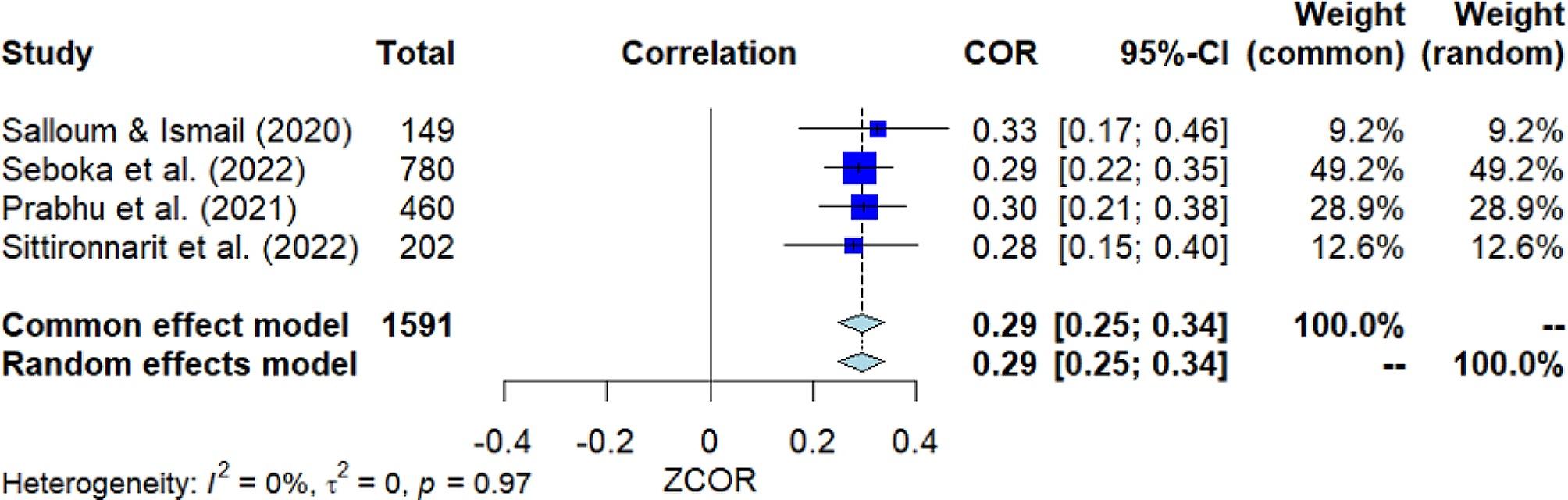
Forest plot for the correlation between seeking help from mental health professionals and MHL

Four studies investigated the correlation between seeking help from mental health professionals and MHL (Fig. 5). The pooled correlation resulted in a significant positive outcome, with *r* = 0.29 [95% *CI*: 0.25, 0.34]. The *Q*-test indicated considerable heterogeneity among the studies (*Q*(3) = 0.27, *p* = 0.966, *I*^2^ = 0.0%). Nevertheless, Egger’s test did not show significance, suggesting no presence of publication bias.

### Attitudes toward help-seeking

**Fig. 6:**
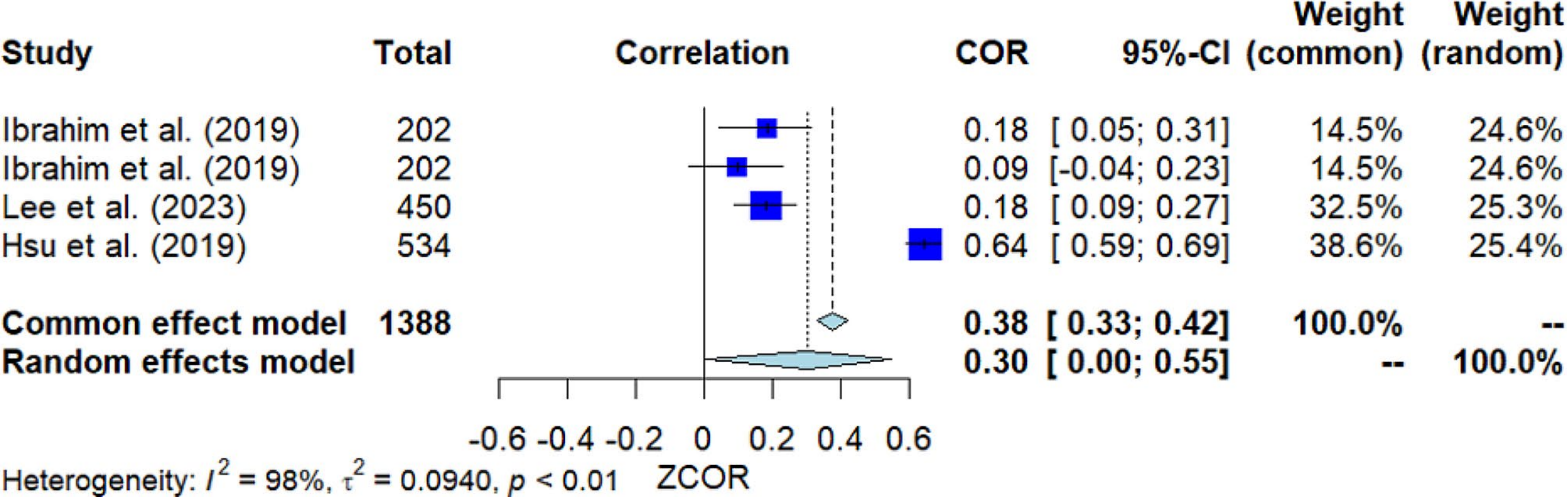
Forest plot for the correlation between attitudes toward help-seeking and MHL

Four studies investigated the correlation between attitudes toward help-seeking and MHL (Fig. 6). The pooled correlation resulted in a significant positive outcome, with *r* = 0.30 [95% *CI*: 0.00, 0.55]. The *Q*-test indicated considerable heterogeneity among the studies (*Q*(3) = 120.29, *p* = 0.000, *I*^2^ = 97.5%). Nevertheless, Egger’s test did not show significance, suggesting no presence of publication bias.

### Social support

**Fig. 7:**
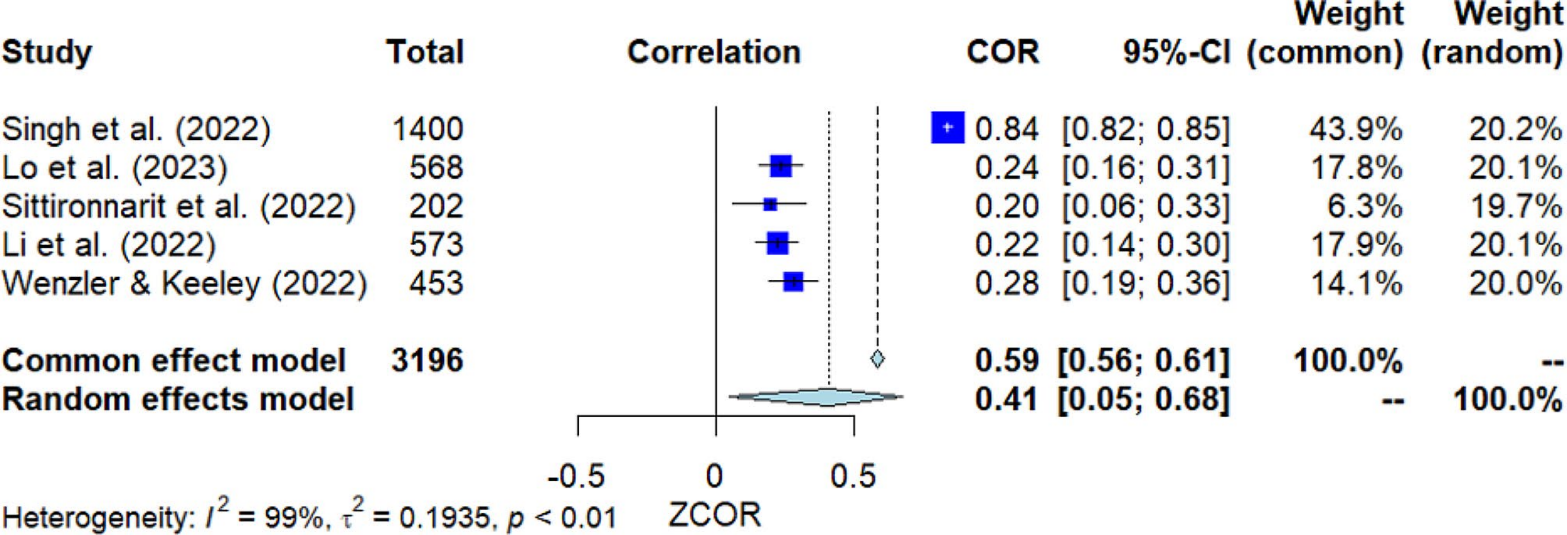
Forest plot for the correlation between social support and MHL

Five studies investigated the correlation between social support and MHL (Fig. 7). The pooled correlation resulted in a significant positive finding, with *r* = 0.41 [95% *CI*: 0.05, 0.68]. The *Q*-test indicated substantial heterogeneity among the studies (*Q*(4) = 748.26, *p* = 0.000, *I*^2^ = 99.5%). However, Egger’s test did not show significance, indicating no presence of publication bias.

### Positive psychological states

**Fig. 8:**
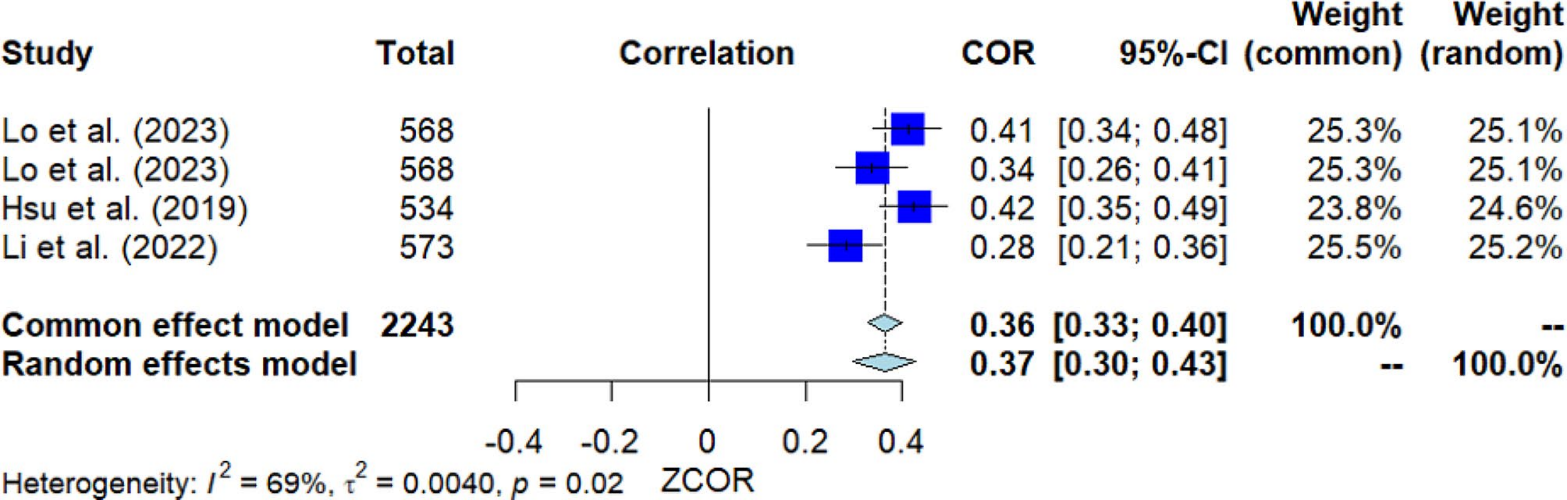
Forest plot for the correlation between positive psychological states and MHL

Four studies investigated the correlation between positive psychological states and MHL (Fig. 8). The pooled correlation resulted in a significant positive outcome, with *r* = 0.37 [95% *CI*: 0.30, 0.43]. The *Q*-test indicated considerable heterogeneity among the studies (*Q*(3) = 9.75, *p* = 0.021, *I*^2^ = 69.2%). Nevertheless, Egger’s test did not show significance, suggesting no presence of publication bias.

### Receiving mental health training

**Fig. 9:**
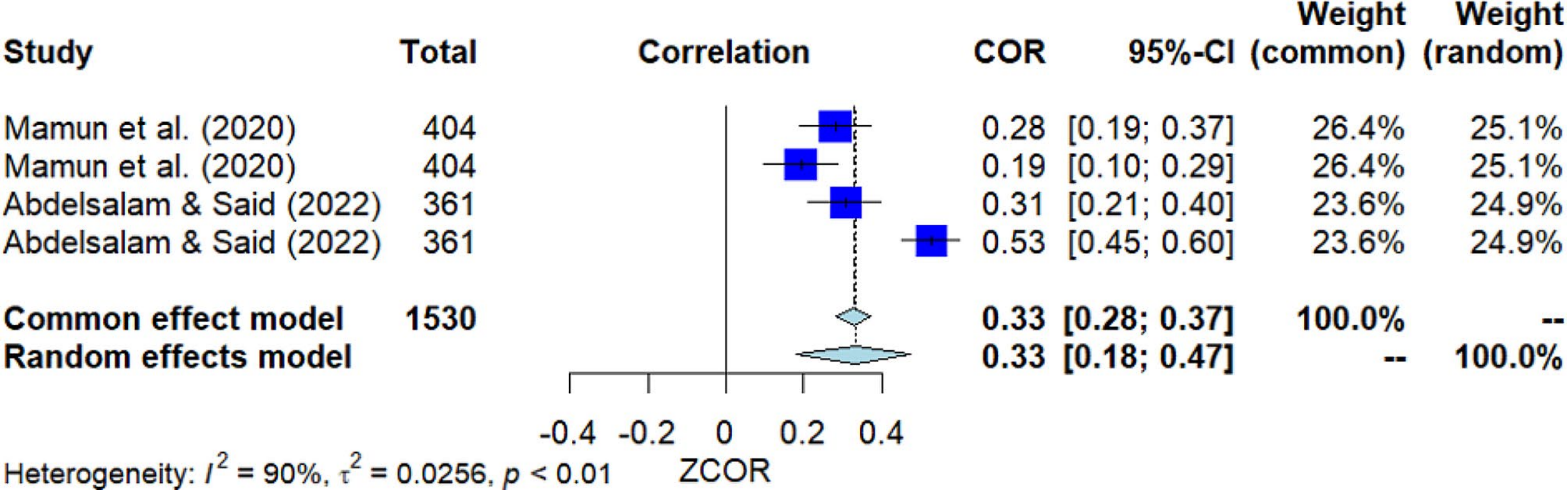
Forest plot for the correlation between receiving mental health training and MHL

Four studies investigated the correlation between receiving mental health training and MHL (Fig. 9). The pooled correlation resulted in a significant positive finding, with *r* = 0.33 [95% *CI*: 0.18, 0.47]. The *Q*-test indicated substantial heterogeneity among the studies (*Q*(3) = 31.57, *p* = 0.000, *I*^2^ = 90.5%). However, Egger’s test did not show significance, suggesting no presence of publication bias.

### Psychological distress

**Fig. 10:**
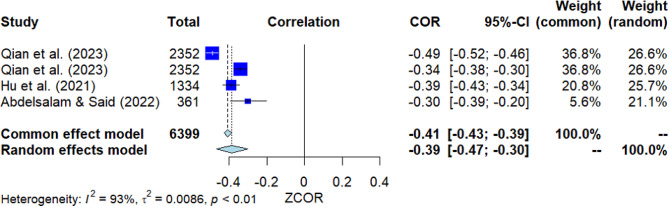
Forest plot for the correlation between psychological distress and MHL

Four studies investigated the correlation between psychological distress and MHL (Fig. 10). The pooled correlation resulted in a significant negative outcome, with *r* = -0.39 [95% *CI*: -0.47, -0.30]. The *Q*-test indicated substantial heterogeneity among the studies (*Q*(3) = 45.56, *p* = 0.000, *I*^2^ = 93.4%). However, Egger’s test did not show significance, suggesting no presence of publication bias.

### Impact of modifiable predictors on MHL

The effect size for each modifiable predictor varied between − 0.39 and 0.41. Social support emerged as the most influential predictor, positively impacting MHL, followed by positive psychological states, received mental health training, attitudes towards help seeking, seeking help from mental health professionals, and self-efficacy. In contrast, psychological distress was identified as the most significant predictor with a negative impact on MHL, followed by stigma towards professional help. However, the effect size for stigma towards mental illness was not statistically significant.

## Discussion

In this systematic review, we meticulously examined existing quantitative evidence on modifiable predictors influencing MHL in educational settings. Our primary goal was to identify areas for improvement and lay the foundation for robust early interventions in mental health within educational contexts. Our findings highlighted consistent associations between MHL and various factors, including stigma toward professional help, self-efficacy, attitudes toward help-seeking, social support, positive psychological states, receiving mental health training, and psychological distress. Importantly, no significant association was found between stigma toward mental illness and MHL.

### Stigma toward professional help

Our analysis confirmed a negative association between stigma toward professional help and MHL, aligning with prior research [53, 54]. Individuals with stigmatizing views on seeking support from mental health professionals exhibited lower MHL in educational contexts. This resistance to seeking professional health assistance highlights the necessity for tailored interventions within educational institutions aimed at enhancing MHL and destigmatize professional help-seeking behaviors. Anti-stigma interventions, such as psychoeducation and contact-based learning, have proven effective in reducing stigma and enhancing MHL in educational environments [55]. By implementing targeted interventions that address stigma toward professional help, educational institutions can play a pivotal role in creating an atmosphere where mental health is prioritized. These interventions should go beyond merely providing information; they should actively work to change perceptions and attitudes related to mental health support. Through comprehensive mental health education covering various mental health conditions, symptoms, and available treatments, as well as the launch of stigma reduction campaigns that challenge negative stereotypes surrounding mental health issues, institutions can contribute to breaking down barriers to seeking professional help, ultimately fostering a more mentally health-literate community.

### Self-efficacy

The significant positive relationship between self-efficacy and MHL highlights the importance of self-efficacy in seeking help within educational settings. Individuals with higher self-efficacy are more likely to possess improved MHL, especially in seeking assistance for mental health-related issues. Nurturing self-efficacy in seeking help becomes a crucial aspect of comprehensive mental health education in educational environments [56, 57]. Self-efficacy refers to an individual’s belief in their capacity to execute actions necessary to achieve specific goals [58]. In the context of mental health, fostering self-efficacy involves instilling confidence in individuals to take proactive steps in addressing their mental health needs. When individuals feel capable and confident in their ability to seek assistance, they are more likely to take proactive steps to address mental health challenges. However, barriers like lack of self-efficacy may impede individuals from seeking help, hindering progress towards MHL and well-being. Integrating self-efficacy-focused interventions into mental health education curricula empowers individuals to actively engage with mental health resources, contributing to a supportive and mentally health-literate educational community. This cultivates an environment where mental health is openly discussed, reducing stigma and promoting a culture of support and understanding.

### Seeking help from mental health professionals

The significant positive correlation between seeking help from mental health professionals emphasizes the importance of encouraging help-seeking behaviors in comprehensive mental health education. Consistent with previous studies [37, 59], individuals who actively seek assistance from mental health professionals are more likely to possess a better understanding of mental health issues, their own mental well-being, and the available avenues for support and treatment. Encouraging help-seeking behaviors can take various forms within the educational environment. Educational programs and interventions aimed at supporting students and staff in reaching out to mental health professionals play a crucial role in fostering proactive engagement with mental health. One potential barrier to implementing these changes is individuals’ reluctance to seek assistance from mental health professionals, often due to stigma, fear of judgment, or lack of awareness about available resources. These initiatives may involve workshops covering topics such as recognizing mental health concerns, understanding available resources, and navigating the mental health support system. Additionally, providing easily accessible resources on mental health support services and self-help materials through online platforms and educational support centers ensure individuals have the necessary information to seek assistance when facing mental health challenges. Such programs contribute not only to individual well-being but also to the broader goal of developing a more mentally health-literate educational community [60, 61].

### Attitudes toward help seeking

The significant positive correlation between attitudes toward help-seeking and MHL reveals insights into the dynamics of MHL within educational contexts. Consistent with previous studies [35, 62], positive attitudes toward seeking help are associated with higher levels of MHL. Understanding and cultivating these positive attitudes within educational institutions can play a pivotal role in shaping a more mentally health-literate community [55, 63]. Educational interventions and programs play a crucial role in cultivating positive attitudes toward help-seeking. One potential barrier in this scenario revolves around individuals who already possess positive attitudes toward help-seeking. They might not see the immediate need to actively engage in seeking further assistance or resources, as they perceived themselves as already well-informed about mental health issues. Initiatives that aim to reduce stigma, dispel myths surrounding mental health, and promote positive beliefs about seeking assistance are integral components of mental health education. These efforts contribute to the creation of a supportive and understanding environment, encouraging individuals to actively engage with mental health resources and seek help when needed.

### Social support

Our analysis uncovered a significant positive correlation between social support and MHL, highlighting the influential role of interpersonal relationships in shaping individuals’ mental health awareness. Consistent with previous studies [38, 64], individuals receiving social support tend to have higher levels of MHL. Understanding the positive impact of social support on MHL opens avenues for educators and policymakers to consider targeted interventions that foster a supportive social environment within educational contexts. Potential barriers include a lack of resources; educational institutions may encounter limitations in terms of funding, staffing, or infrastructure to develop and sustain programs. Additionally, there may be resistance from certain stakeholders within the educational community, such as administrators, teachers, or students, who may resist changes in policies or practices. One effective strategy involves enhancing peer relationships through structured programs and activities. Peer support initiatives, mentorship programs, and community-building activities create opportunities for students to connect with one another, share experiences, and offer mutual support. These initiatives not only contribute to students’ overall well-being but also play a crucial role in fostering a mentally health-literate educational community [65, 66].

### Positive psychological states

The significant positive correlation between positive psychological states and MHL emphasizes the substantial contribution of these characteristics to the development of a more mentally health-literate educational community. Consistent with previous studies, individuals with positive psychological states tend to have higher levels of MHL [67, 68]. The recognition of the positive impact of these states provides educational institutions with valuable insights to advocate for the integration of mental health education into curricula. Emphasizing the cultivation of positive psychological states becomes a key aspect of a comprehensive approach to MHL [68]. Designing curriculum modules focused on a positive psychology approach represents a proactive and empowering strategy for enhancing MHL in educational contexts. These modules provide an opportunity for students to explore and develop positive psychological states such as resilience, mindfulness, and enthusiasm. By participating in engaging activities such as group projects, role-playing scenarios, and case studies, along with reflective exercises like journaling and self-assessments, and through fostering open discussions in class, students can enhance their understanding of these states and apply them in real-life situations. This not only contributes to improved MHL but also equips students with valuable life skills that extend beyond the educational setting. One potential barrier is the resistance to adopting new teaching methodologies or content. Educators or institutions may be accustomed to traditional approaches to mental health education and may be hesitant to embrace newer, positive psychology-focused methods. Overcoming these barriers may require comprehensive training and professional development for educators, clear communication about the benefits of positive psychology approaches, and collaboration with relevant stakeholders to ensure buy-in and support for implementation.

### Receiving mental health training

The significant positive correlation between receiving mental health training and MHL highlights the pivotal role of training programs in fostering a mentally health-literate community. Advocating for structured mental health training programs in educational settings, covering topics such as mental health awareness, coping strategies, and destigmatization, can equip students and staff with the knowledge and skills to navigate mental health challenges effectively [13, 69, 70]. Advocating for the integration of mental health training into educational curricula recognizes the transformative impact these programs can have on the overall well-being of the community. It aligns with a proactive approach to mental health, emphasizing prevention, early intervention, and the cultivation of a supportive culture. One potential barrier is the challenge of prioritizing mental health education among competing academic and institutional priorities. Educational institutions may face pressure to allocate resources and time to other areas of study, potentially relegating mental health training to a lower priority. Additionally, there may be resistance from stakeholders who perceive mental health training as unnecessary or unrelated to academic achievement. Addressing this barrier requires effective advocacy efforts to demonstrate the importance of mental health training in promoting overall well-being and academic success. This may involve providing evidence-based research on the benefits of mental health education, engaging with decision-makers to gain their support, and fostering a culture that values mental health and wellness within the educational community. Acknowledging the role of training in curricula signals a commitment to creating an informed, empathetic, and mentally health-literate educational environment.

### Psychological distress

The significant negative correlation between psychological distress and MHL sheds light on the intricate relationship between psychological distress and the ability to comprehend and respond to mental health issues. Previous studies [71, 72] suggest that students experiencing higher levels of psychological distress may face challenges in understanding and addressing mental health concerns. Integrating mental health support services, coping mechanisms, and stress management strategies into educational programs contributes to a comprehensive approach to fostering MHL, emphasizing sensitivity to students’ mental well-being. For example, students facing elevated psychological distress may encounter barriers to engaging with and comprehending mental health information [73]. The emotional and cognitive burden associated with distress can impede the capacity to absorb and apply concepts related to mental health, potentially leading to a reduced ability to recognize signs of distress in oneself or others. Furthermore, individuals experiencing psychological distress may be less inclined to seek help or participate in mental health programs due to feelings of shame, embarrassment, or stigma surrounding their mental health challenges. They may also fear judgment from peers or staff, leading them to withdraw or avoid seeking support altogether. This emphasizes the need for educational institutions to be proactive in addressing the mental health needs of students, especially during periods of heightened psychological distress.

Each modifiable predictor identified can be theoretically grounded in the Ecological Systems Theory [74], which explores the dynamic interplay between individuals and their social, cultural, and environmental surroundings. Within this theoretical framework, modifiable predictors of MHL in educational contexts––such as social support networks, institutional policies, and cultural attitudes toward mental health––can be analyzed. This perspective allows for an examination of how these factors shape individuals’ MHL within educational settings. By adopting this approach, researchers and educators can gain a comprehensive understanding of the factors influencing MHL in educational contexts., facilitating the development of interventions that target modifiable predictors across multiple levels of the ecological system. Ultimately, this approach aims to foster positive mental health outcomes among students and enhance the overall well-being of the educational community.

## Strengths and limitations

The study highlights the contextual relevance of MHL within educational settings, acknowledging the intricate interplay of social, emotional, and intellectual development in schools, colleges, and universities. This contextualization not only enriches the analysis but also provides actionable insights for educational interventions, addressing a research gap by specifically focusing on modifiable factors related to MHL within educational environments. Despite these valuable contributions, several limitations warrant consideration. First, certain factors exhibited high heterogeneity, potentially influencing the robustness and generalizability of the meta-analysis findings. The diverse nature of educational contexts, including variations in cultural norms, educational systems, and demographic characteristics, may contribute to this heterogeneity. The study refrained from conducting a subgroup analysis due to the limited number of original studies, highlighting the need for future research to explore how contextual differences impact the associations between predictors and MHL. Second, the heterogeneity of the study population, encompassing a diverse range of participants from college students to teachers, emerges as a noteworthy limitation. While this diversity mirrors the intricate tapestry of educational environments, it introduces potential sources of variability that could impact the precision and generalizability of the study’s findings. Third, the majority of studies were conducted in middle-income countries, posing a challenge to the broad applicability of the findings across different socioeconomic contexts. Educational systems, cultural attitudes toward mental health, and the availability of mental health resources can vary significantly between countries and regions. Future research should strive for a more globally representative sample to enhance the external validity of the study’s conclusions. Fourth, potential biases in study selection might have influenced the overall bias, particularly if certain studies were omitted due to specific characteristics like educational contexts. Future research should consider broader criteria for selecting studies, including a wider array of educational settings and populations. This would help ensure that the results are more inclusive and applicable to diverse populations. Fifth, there is significant variability in how MHL is measured within educational contexts. Different studies employ diverse assessment tools, resulting in inconsistencies and challenges in synthesizing findings across studies. Future research should prioritize the development and validation of standardized measurement tools tailored specifically for assessing MHL in educational contexts. Finally, the study is based on correlational data, emphasizing the need for prudence when deducing a cause-and-effect connection between modifiable predictors and MHL. To characterize this association as causal, more robust research methodologies are needed, incorporating longitudinal or repeated assessment of MHL alongside potentially modifiable predictors. Additionally, longitudinal studies to assess the impact of interventions derived from modifiable predictors on MHL over time are also essential.

## Conclusions

This systematic review aimed to fill the existing research gap concerning modifiable predictors influencing MHL in educational settings. The study identified and examined potential predictors associated with MHL, including stigma toward professional help, self-efficacy, attitudes toward help seeking, social support, positive psychological states, receiving mental health training, and psychological distress. By addressing these factors, educational institutions can strive to cultivate communities that are adept in mental health, fostering an environment characterized by empathy, understanding, and proactive engagement in addressing mental health issues. The implications extend beyond individual well-being, reaching into the establishment of inclusive, safe, and supportive learning environments within educational institutions. The study’s findings serve as a foundation for future research, policy development, and the implementation of practical strategies aimed at enhancing MHL in diverse educational settings.

## Data Availability

No datasets were generated or analysed during the current study.
